# Creating ethics guidelines for artificial intelligence and big data analytics customers: The case of the consumer European insurance market

**DOI:** 10.1016/j.patter.2021.100362

**Published:** 2021-10-08

**Authors:** Martin Mullins, Christopher P. Holland, Martin Cunneen

**Affiliations:** 1 University of Limerick, Kemmy Business School, Limerick, Ireland; 2 Loughborough University, Loughborough LE11 3TU, UK

**Keywords:** data analytics, insurance, InsurTech, AI, AI ethics, AI regulation, AI governance, risk

## Abstract

The European Union (EU) has a strong reputation and track record for the development of guidelines for the ethical use of artificial intelligence (AI) generally. In this paper, we discuss the development of an AI and ethical framework by the European Insurance and Occupational Pensions Authority (EIOPA), for the European insurance market. EIOPA's earlier report on big data analytics (EIOPA, 2019) provided a foundation to analyze the complex range of issues associated with AI being deployed in insurance, such as behavioral insurance, parametric products, novel pricing and risk assessment algorithms, e-service, and claims management. The paper presents an overview of AI in insurance applications throughout the insurance value chain. A general discussion of ethics, AI, and insurance is provided, and a new hierarchical model is presented that describes insurance as a complex system that can be analyzed by taking a layered, multi-level approach that maps ethical issues directly to specific level(s).

## Highlights

• A socio-ethical framing of BDA, AI, and ML applications in the insurance pipeline
• Analysis of AI in insurance operations regarding customer behavioral data

## The bigger picture

Big data analytics and increasingly sophisticated forms of artificial intelligence (AI) and machine learning applications are now part of the catalog of tools and methods that inform the insurance industry's back-end and front-end operations. Insurance products and services are important to support economic and societal development. From commercial property insurance, vehicle insurance, and personal health insurance, availability and access to affordable insurance products are key to society and citizens. That said, insurance products and services can only be accessible if a sustainable balance is achieved between the pricing of policies, operational costs, and claims. AI and machine learning have already proved valuable to the insurance industry in sustaining this balance. However, with the increased use of AI and machine learning, new risks and ethical questions are introduced to the insurance pipeline. Accordingly, there is a need to investigate the ethical contexts and questions of data use in insurance innovation and highlight the emerging tensions and concerns where possible. The paper combines the expertise of insurance experts and an AI ethics expert to assess and better understand some of the ethical ground relating to the tensions concerning how the insurance industry uses big data analytics, AI, and machine learning methods to sustain its commercial operations.

## Teaser

The research aims to provide a deep dive into the increasing ethical questions and contexts relating to the insurance industry's sophisticated use of big data analytics, AI, and machine learning to sustain and develop its products and services. While the commercial opportunities are clear, there are questions of what costs the commercial benefits have in terms of how data are used and if the use leads to further tensions regarding privacy, surveillance, and profiling.

## Introduction

Over the past 12 months, two of the paper's authors have been working with the European Insurance and Occupational Pensions Authority (EIOPA) expert group on digital ethics on creating a set of guidelines on the ethical use of artificial intelligence (AI) and big data analysis on the part of the insurance industry. The report was published in June 2021.^1^ The group is made up of representatives from the insurance industry, non-governmental organizations (NGOs) along with trade unions and members from academia. In terms of previous work in the field, the group had one of its starting points, the EIOPA report on big data analytics (BDA) in motor and health insurance.^2^ Debates in the group have centered on how to ensure that consumer or citizen rights are protected while at the same time allowing the insurance industry to benefit from new technologies and techniques around the processing of data and big data, which often involves machine learning and AI. More generally, the clear trade-offs between privacy concerns and the ability of firms to innovate will determine the nature of the roll-out of AI.^3^ In other words, we take the essential role of the group to weigh up the balance between innovation in this field with the potential for negative externalities. During the last year or so, members of the group have pointed to many upsides of BDA. These have included improvements in road safety offered by the use of telematics data^4^, ^5^, ^6^, ^7^, ^8^ as well as how improved analytics have allowed groups that were previously difficult to insure to be brought under the insurance umbrella, by offering new usage-based insurance based on telematics.^9^, ^10^, ^11^, ^12^, ^13^ We have also had interventions from those members concerned over the consequences of the roll-out of this new technology for vulnerable groups and for the possible impacts in terms of financial inclusion.^11^ There are also broader concerns in the research domain speaking to the commercial use applications of user data and the tensions with privacy concerns,^14^, ^15^, ^16^, ^17^ profiling,^11^^,^^18^ and user perception.^19^ There have also been extensive debates on the potential for opacity in these novel risk assessment techniques and the need to aim for explainability.^20^^,^^21^ These are just a small sample of the discussions that took place and speak to existing and emerging narratives of disruption, insurance solidarity, and social perception and concerns.^22^

Our very preliminary position at this juncture is one where we accept that there are significant operational, safety, user, and broader societal benefits and risks posed by the introduction of BDA and AI/machine learning into the insurance industry.^23^^,^^24^ Thus, this paper focuses on the insurance industry, which is a key end user of big data and has a very bespoke and profound set of social relations underpinning its practice. Accordingly, any ethical analysis needs to be grounded in the socio-technological detail of the type of relations created by this new set of practices and the availability of new data-focused applications. At different points in the insurance value chain, tensions can be captured in terms of ethical dilemmas that are more or less acute. One possible way to navigate what EIOPA posit as “digital ethics” across insurance services is to contextualize AI and digital applications as socio-technological relations. This is already emerging in the cases of health and motor insurance by reference to a form of relational ontology or relationality between commercial data use and citizen data use.^25^ This requirement to understand the relationality has been implicit in the structuring of work within the expert group, with different sub-groups tackling distinct areas or business lines of insurance. To give an example, there are differences in the relations that exist in the area of health insurance from that of domestic home insurance lines of business. While both are united in a commercial application, there are diverse relations that remain unclear and in need of investigation. This allows for a more apt capture of the fecundity of the various cases that present themselves. The pace of innovation is presenting many changes in terms of capturing and anticipating societal relations. Ethics is also adapting to this challenge as presented in the move to relational ethics and the move toward the empirical in debates on technological ethics, as identified by Vallor (See Chapter 1 of Vallor, S., 2016. Technology and the virtues: A philosophical guide to a future worth wanting. Oxford University Press).^26^

There can be little doubt that the BDA will affect commercial insurance operations and has the potential to bring about dramatic change and disruption by changing the paradigm of traditional information regimes in which insurance operates.^27^, ^28^, ^29^ Access to large caches of personal information on existing and potential clients offers insurers a way of better managing long-present issues around information asymmetry and moral hazard.^30^, ^31^, ^32^, ^33^, ^34^ Both of these become increasingly complex as datafication and insights continue to provide more accurate risk pricing and product development.^32^^,^^35^ Perhaps the most immediate impact will afford insurers, and indeed state authorities more generally, more effective tools to counter insurance fraud and to address the phenomenon of “compensation culture” that exists in some European jurisdictions.^36^, ^37^, ^38^, ^39^, ^40^, ^41^, ^42^

More generally, emerging data analytics innovations have had a profound impact on the nature of information flows across society and the economy, both in the manner that information is processed and indeed how this processing is communicated to other stakeholders and, most importantly, to end users.^43^ Behavioral analytics and image processing, alongside protocols around sentiment analysis and event detection, have transformed relations between citizens and corporate entities as well as state bodies.^44^ As a result, new dynamic socio-technological relations have evolved that pull in state, commercial actors, and citizenry into a new paradigm that seeks to sustain the social solidarity benefits of insurance while also respecting civil liberties and rights.^45^, ^46^, ^47^, ^48^ With these novel information flows come a new set of risks that require new agreements between stakeholders to provide balance.^14^ This can only be achieved and managed by interrogating new governance regimes^49^^,^^50^ informed by the risks.^14^

Shoshanna Zuboff in her book, *Surveillance Capitalism*, sets out the profound dangers attendant to the so-called data economy.^51^ The book’s focus is on “big tech,” but she does touch upon the practice of insurance as an important example^52^ of the logic to the new paradigm of surveillance capitalism.^53^ Her arguments center on how the technologies of surveillance have changed the relationship between citizens and the capital and how this change threatens long-held and precious ideals of human dignity, freedom, and autonomy.^51^ The advent of the digital economy has afforded many industries an unprecedented opportunity to utilize BDA and AI in order to process information about clients, customers, and users. This has prompted many international institutions and national governments to produce reports and white papers on the ethical use of digital technologies (These include the EU, the Organisation for Economic Co-operation and Development (OECD), and a number of member state governments (Falk & Unterlass, 2006; "The knowledge-based economy," 2020)). There can be no doubt that, in any such critique of the data economy, be it from Zuboff's perspective or from a more Foucauldian position,^54^, ^55^, ^56^ insurance is a key piece of the architecture. Indeed, for many years, Foucauldian scholars pointed to the importance of a risk-based discourse and disciplinary power possessed by the insurance industry.^54^^,^^55^^,^^57^ Under the general heading of “biopolitics,” works by Mike Dillon and others from the 1990s onward have highlighted the asymmetrical power relations between insurance companies and their clients.^58^ With such a set of power relations comes responsibility and the need for ethical oversight (Dillon, M., 2008. Underwriting security. Security dialogue, 39(2–3), pp.309–332).

More recently, discourses originating with Bentham's panopticon or Foucault's *Discipline and Punish* can be supplemented by thinkers who interrogate the phenomena of AI through an applied ethics lens.^59^, ^60^, ^61^, ^62^, ^63^, ^64^, ^65^ While such authors are able to bring to bear a deep knowledge of AI and machine learning, the set of concerns remain quite similar. Notions of rights, agency, consent, surveillance, privacy, and human autonomy remain to the fore.^14^, ^15^, ^16^^,^^52^^,^^66^^,^^67^ There is also a growing discourse around the risks relating to how populous scales of adoption concerning data and machine intelligence applications introduce further downstream unknowns such as societal and behavioral impacts relating to "design-based control."^16^^,^^68^^,^^69^

## Results

### The case of insurance

The centrality of insurance to the modern economy and indeed to modernity itself cannot be overlooked (See Ferguson, N., 2008. The ascent of money: A financial history of the world. Penguin. And Lupton, D., 2006. Risk and governmentality. The sociology of risk and gambling reader, pp.85–99). Within the academy, faculties with specialists in insurance are somewhat few and far between. That said, both sociologists and historians have made a strong case for the centrality of insurance in any wider analysis of society (Ewold, F., 1991. Insurance and risk. The Foucault effect: Studies in governmentality, 197, 210). As a practice, it has its own set of ethics, and indeed, at its core, it is a risk-sharing platform with welfare outcome as a central objective. Addressing digital and specifically, data ethics for the insurance industry is an increasingly important and necessary task. The operation of the insurance market has important economic and welfare functions for wider society and can generate both positive and negative externalities. In terms of social inclusion, life, health, and non-life insurance lines all play an important role. Therefore, it is advantageous to engage in reflexive foresight to, where possible, anticipate the emerging socio-technological tensions within the emerging data-centered insurance ecosystem.

That said, given the centrality of the insurance industry to the life of European Union (EU) citizenry, it points to a need for a bespoke socio-technological contextual approach to digital and data ethics as it pertains to the insurance profession. The digital economy introduces new classes of risk around the consumer and, in many instances, threatens information asymmetry between client and insurer (Hamilton, 2018). From a regulatory perspective, there are risks around fairness, non-discrimination, and how data are recycled for other commercial gains.^70^ The challenge for regulators and supervisors alike resides in allowing the European insurance sector to take advantage of the innovation offered by the digital economy while at the same time protecting the interests of consumers and citizens (From Bernardino, G., 2020. Challenges and opportunities for the insurance sector in Europe. In Annales des Mines-Realites industrielles (No. 1, pp. 99-102). FFE).

The precautionary principle is also in play here.^71^, ^72^, ^73^ Discussions with the EIOPA Expert Group on Digital Ethics mirrors debates that have been taking place in academia over the past three decades, with some members’ positions being close to that of Cass Sunstein^74^, ^75^, ^76^, ^77^, ^78^, ^79^ and others Powell^80^ in arguing that the regulation has the potential to damage innovation,^81^ and others taking a more precautionary stance to data-centered business models.^82^ Many of the issues thrown up by the use of big data by insurers may have unwelcome long-term social consequences. For example, there is the potential that the adoption of more granular datasets by insurers may undermine ideas of risk sharing in terms of positive distribution and social solidarity and the belief in fairness that underpins it.^83^ There are also some justified concerns over the advent of surveillance regimes whereby BDA might be used to exclude certain cohorts from access to important financial instruments inherent to insurance products and services. This also speaks to the issue of non-discrimination, fairness, inclusion, and access. The impact on human autonomy and subjectivities around freedom are important concerns and cannot be simply dismissed as dystopian fantasies. Where the precautionary principle leads us in the context of such uncertainty may be an ethic of care and protection where we aim to safeguard post-war European values. With regard to digitalization and insurance, we are in the early stages of adoption, and hence this report is a timely intervention in the debates around fairness and non-discrimination. The debates on the value of the precautionary principle versus the innovation principle rage on, as do discussions that reflect these two positions, and the expert group was no different in this regard.^84^, ^85^, ^86^

With BDA and AI in insurance, we find ourselves in a classic pacing problem dilemma.^87^^,^^88^ We see this across and range of economic activities where the speed of technological developments is such that the regulatory and legal responses cannot keep pace. It is a risk governance problem that is widely cited and implies the need for a response from public policymakers, industry and civil society alike. To date, much of the focus has resided in material science and biotechnology, but the pacing problem is now embedded in financial services. In the absence of clear sets of rules or a well-established body of law, be it hard or soft, there is a strong emphasis on the development of a set of ethics to mitigate conduct risk. As AI has become a standard evolutionary path for the insurance industry, laws struggled to keep up with the pace of digital innovation, and existing regulations are often miscalibrated and misaligned to present risks. The scale and ease with which AI can be used and analytics can be conducted today add new ethical challenges. In this context, compliance could on some occasions be confused with complacency; that is, if ethical relations are not effectively elucidated and not brought into consideration, the risks of the innovations and applications are dramatically increased. In this way, the import of ethical elucidation is increasingly important.

### Specific ethical issues pertaining to BDA and AI insurance

Insurance is about the technology of risk,^55^^,^^57^^,^^89^, ^90^, ^91^ which is inherently a data analysis and prediction problem. This is why BDA^2^ and the use of AI is strategically important to the insurance industry for the calculation of risk related to pricing and underwriting. AI is a general purpose technology (GPT),^92^ so, in addition to its use as a sophisticated tool for prediction,^3^ related to risk assessment, it is also used throughout the customer journey from new customer acquisition to e-service, claims, renewals, and product development. Hence, it is sometimes vital to contextualize use contexts with an ethical lens because it is only when the ethical problem is viewed within a meaningful business and social context that the current, and potential future, direction of AI technology can be explored and debated in a sufficiently detailed and nuanced manner. An overview of AI applications in insurance is shown in Table 1. These applications are numerous and represent the growing ecosystem of AI within the insurance industry. The applications point to a variety of front-end and back-end operations, from prediction to claims handling.

**Table 1:** An overview of BDA and AI applications in insurance

Insurance value chain	Business process	AI systems and big data	Main ethical issues
Market research and product design	market research	customer and survey data to inform new product development	Market exclusion for vulnerable and inherently unprofitable market segments
	product development	a/b testing of new digital and insurance service designs	bias toward the existing customers of incumbent firms
Pricing and underwriting	Risk assessment	(a) traditional data sources; e.g., customer information, exposure data, and loss data to estimate risk profile for groups of customers (b) new forms of big data; e.g., behavioral data, Internet of Things, telematics, personal tracker data and Global Positioning System to inform behavioral analytics based on advanced machine learning^1^	customer data increase the value of algorithms, but the benefits may not be shared with them micro-segmentation may lead to the loss of insurance pooling and exclude small groups of high-risk customers (See Cevolini, A. and Esposito, E., 2020. From pool to profile: Social consequences of algorithmic prediction in insurance. Big Data & Society, 7(2), p.2053951720939228. They too make the case that developments in big data in insurance pose a threat to the pooling paradigm) an undermining of the risk-sharing paradigm that has historically underpinned insurance and a commensurate decline in social solidarity privacy and surveillance issues alongside concerns over citizen autonomy re-selling of data and personal data commodification
	pricing	(a) machine learning to develop pricing models that are based on customer groups (b) micro-segment/personalized pricing based on individual behavioral data	algorithmic bias and use of non-risk data in pricing pricing based on micro-segmentation is difficult to explain because of the number of variables and algorithmic complexity social solidarity concerns information asymmetry and related explainability concerns market exclusion from high pricing related to extremely high risk of specific individuals and micro-segments
Sales, distribution, and marketing	sales and marketing	digital marketing techniques based on the dynamic analysis of online search behavior	potential to coerce customers into buying unnecessary or expensive insurance and the exclusion of vulnerable groups
	customer acquisition and retention	virtual assistant and chatbots that utilize NLP and insurance ontologies to support communication	potential for automation of incorrect or harmful advice atomization of offerings means that product offerings become financial advice biased selection of new customers
	CRM	CRM based on sophisticated communication and analytics that are based on advanced models of consumer behavior and switching to new competitors	exploitation of customer characteristics such as inertia and lack of knowledge lock-in effects on customers may reduce their propensity to search and find better products and services, and risk of biased and/or incorrect advice
	behavioral analysis of customers	behavioral risk analysis in insurance sectors such as health and automotive	unintended consequences such as customers gaming the system may lead to unsafe behavior, e.g., exercising when feeling ill, and being distracted by insurance safety advice when driving monitoring could develop into surveillance increases information asymmetry between consumers and insurance companies
E-service	insurance policy management	robotic process automation	unfair bias in the rules-based processing of policy documents, claims, and e-service
	customer self-service through multiple channels	NLP, voice recognition, insurance ontology maps and virtual robots to facilitate customer self-service and offer advice	excludes or discriminates against customers with poor technology knowledge and skills, and potential for incorrect advice and guidance that could result in unsuitable policy amendments and purchases
	customer complaints	automated resolution of a high proportion of customer complaints through all marketing channels	risk of algorithmic bias in the resolution of a complaint to the detriment of specific customer segments
Risk mitigation and claims management	risk mitigation to reduce claims	AI-based safety warning systems such as collision detection and automated braking coaching of driving performance and health behavior based on AI risk assessment	the trade-off between individual safety versus the safety of other individuals and groups cannot always be calculated, and could lead to unintended harm from flaws in the safety systems behavioral nudging could lead to loss of individual freedoms and independent decision making, and there is the risk of incorrect advice
	claim processing	AI image recognition to estimate repair costs in household property insurance, business premises, and automotive claims	there is the potential to withhold the true value of the claim; e.g., through the short-term inducement of faster payment automated systems may be opaque to stakeholders
	fraud detection	AI fraud detection to identify anomalies in individual claims and search for fraud patterns across all claims	a false accusation of fraud could result from algorithmic bias based on previous fraudulent claims and therefore disadvantage specific groups risk of profiling based on factors that are beyond the control of the individual

CRM, customer relationship management; NLP, natural language processing.

**Table 2:** A hierarchical AI, data, and ethics model

Typical ethical problem associated with each hierarchical level	Ethical problem related to the complex system/levels of abstraction
Algorithmic bias: Level 1 AI technology	the problem of algorithmic bias is best understood at the level of AI technology, because this is where the algorithm is designed and trained, and where the intent is captured through a combination of the initial design and training of the algorithm. At this technology level, transparency relates directly to the design of the algorithm and the data that were used to train it. This level is common to all AI technology and applies naturally to an insurance system, where the data can be defined quite easily to a lay person. The design of the algorithm may be more difficult to explain to non-experts but at least the nature of the ethical issues can be positioned as a technical problem, rather than attempting to resolve it by considering the more general, overall system. The array of data used can also be defined clearly at level 1, which is easily understood by customers where even the most complex data from health trackers and telematics can be described and explained in layman's terms. Issues of data compliance such as GDPR with respect to data usage and the utilization of appropriate datasets can also be analyzed at this level
Surveillance: Level 2 AI capabilities	surveillance of an individual is defined as intrusive monitoring and collection of personal information to infer behavioral characteristics. The collection of data for the purpose of insurance in one context could also potentially be misused by gleaning information about the consumer that could be used in unrelated contexts. Of course, the close monitoring, collection, and analysis of personal data can lead to significant benefits; e.g., to identify early warning signs of health problems or to inform risk assessment. However, there is also the real potential for surveillance where the monitoring and associated analytics become intrusive and goes beyond what might be termed reasonable access to data for insurance purposes. The right to protection from discrimination also relates closely to AI capabilities. For example, analytics capabilities could infer data that relates to protected characteristics, which should not use protected characteristics such as gender, race, or religion
Insurance exclusion: Level 3 insurance technology	insurance exclusion that may arise from very fine, granular understanding of risk from massive datasets and AI is an ethical dilemma because, on the one hand, better analysis of risk gives lower pricing for some customers but may actually exclude others from the market altogether. This is an ethical issue that relates most directly to risk, which is a component service on level 3. The nature of insurance means that it should be accessible to all in a society that values inclusion and peace of mind over events that simply cannot be controlled or managed at the level of the individual. Examples of exclusion could be housing that is at particular risk of flooding, or health problems that are uneconomic to insure. From an AI perspective, it is much more likely that the massive datasets and associated AI capabilities mean that insurance technology becomes much more adept at identifying and distinguishing between groups, which means that the potential for exclusion increases in line with this growth in sophistication of AI systems
Human autonomy: Level 4 insurance products and services	human autonomy is a fundamental human right, and, in extremis, it can be argued that a combination of highly detailed surveillance, combined with pro-active interventions on behalf of the insurance firm, may influence human behavior to such an extent that it starts to limit the autonomy of individuals to make their own decisions. Of course, this is a highly difficult and complex issue to assess, but there is clearly a plausible case to answer when one considers the sophistication, scale, and extent of potential insurance policy designs that seek to change customer behavior to the benefit of the insurance firm. Of course, the customer may also benefit, e.g., from increased exercise, safer driving, and better protection of assets, and the issue becomes one of balancing the conflicting needs and rights of stakeholders. The ethical issue of human autonomy is best considered at the product-market level because this is where the customer uses the insurance service and interacts with the insurance firm through a variety of interactive communication devices; e.g., watch, smartphone, Internet, telematics devices, and smart sensors a typical insurance example would be the problem of automated intervention in customers' lives. Simple warning systems of increased heat and emission of particles that warn of a fire are essential in smart buildings. In a driving situation, when pedestrians walk into the road unexpectedly, an automated braking system can react much quicker than a human driver, and this intervention prevents or reduces the amount of physical harm to the people involved. However, when a health tracker moves from what appear to be simple nudges to increase activity from a sedentary individual or to encourage intensive physical activity as part of a health scheme to a more sophisticated and pervasive control mechanism, when does this start to affect human autonomy in an unacceptable manner?

GDPR, General Data Protection Regulation.

**Figure 1:**
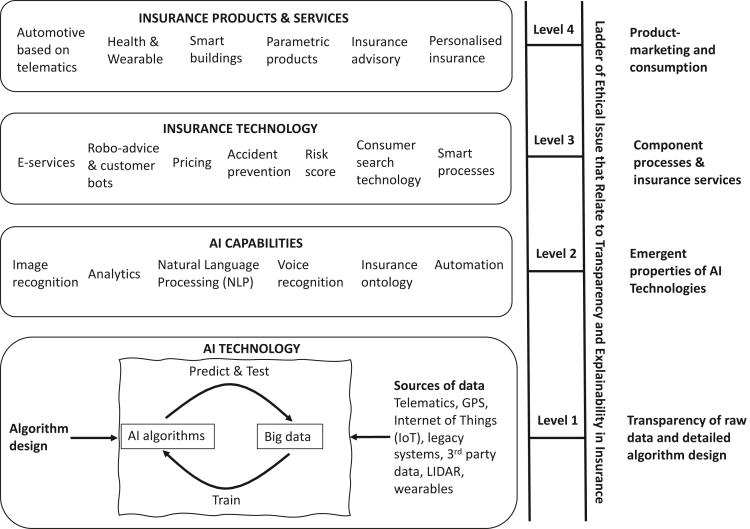
Hierarchical ethics model of AI and big data in insurance markets

The pricing and underwriting function is the obvious starting point to explore BDA and AI applications and ethical issues because this is the essence of insurance and is the gateway for most consumers (and businesses) to insurance markets. A variety of issues related to individual privacy and human autonomy, fairness, and the potential adverse effects on the insurance industry itself are immediately apparent. The use and potential misuse of data is central to these concerns. As to a suitable approach to be adopted by both academic researchers and indeed practitioners, the ethics ladder posited in Figure 1 suggests a method that allows for analysis across the value chain. This would commence with the data sources, move through to the algorithms used, and finish with the actual products offered to consumers. This model speaks to the distinct relational ontologies that exist across the ladder and would allow for the correct stakeholders to be involved in decision making at the right stage.

AI technology exacerbates fundamental data concerns related to privacy and surveillance because the nature of AI algorithms is such that they have an insatiable appetite for more and more data. In a previous era of “small data,” the cost of collecting, storing, curating, analyzing, and sharing the results of sophisticated data analyses across a distributed network of individuals, devices, and organizations acted as a natural brake or control mechanism for data use; the term big data only exists because of radical improvements in computing performance, which makes it economically feasible. Ethical issues that pertain to the intersection between the use of BDA and the pricing/underwriting function go beyond concerns over privacy and surveillance regimes. One of the practices on the part of insurance entities that relate to BDA is so-called non-risk pricing. For example, insurers may use data to gauge your sensitivity to price and your loyalty to an insurer as they use this as a part of a pricing protocol. Thomas^93^ provides a good summary of the practice that re-emerged as a controversy in a number of jurisdictions (See Thomas, R.G., 2012. Non-risk price discrimination in insurance: Market outcomes and public policy. The Geneva Papers on Risk and Insurance-Issues and Practice, 37(1), pp.27–46). In the Irish Republic, the Minister of State in the Department of Finance with special responsibility for financial services, credit unions, and insurance has promised to tackle this practice stating "Insurance companies have a duty to treat their customers fairly and honestly (See https://www.independent.ie/business/targeted-solutions-are-needed-to-help-reform-irelands-insurance-sector-40083374.html)."

Algorithmic bias is also a function of data because the calibration of an AI algorithm is determined by the dataset used to develop it. If the training data have biases against protected characteristics or are simply flawed in other respects, then these characteristics are then built into the design of the new BDA and AI system, resulting in unfair discrimination. In addition to the volume aspect of big data, the concepts of granularity and relatedness are of crucial importance in an insurance context.^94^ Granularity refers to the very fine level of detail that it is possible to collect about someone's characteristics and behavior.^95^

To evaluate AI ethics in insurance, the specific purpose, or business context, for which an AI system is designed, e.g., pricing, to support or enable a business process, and market analysis, defines the context and tells us about the likelihood and potential severity of harm.^96^ A holistic perspective must therefore be taken in any analysis of AI ethics that takes into account the core AI technology of the algorithms, the datasets used in the design and ongoing evolution of the application, and the business/social context in which the technology is applied.

One important line of insurance business where we see a convergence of both ethical and governmental concepts such as fairness, discrimination, and harm is in motor insurance. The potential for conflicts to exist between fairness and discrimination has been signaled by the Test-Achat case, heard by the European Court of Justice in 2011, which made it impermissible for insurance to price on gender grounds.

Since the inception of telematics in motor insurance, this fault line between fairness and discrimination has been widely discussed. In some important ways, the evidential basis that telematics offers for premium settings can be seen as increasing fairness. Indeed, to build on the discussion of the Test-Achat case, when annual mileage is taken into account, gender-based variables decline in importance.^97^ Guillen et al., in their paper on risk analysis, come to similar conclusions; however, the authors also make the case for premium adjustment due to the learning effect of driving long distances.^98^ The granularity of the data introduced by telematics seems to preclude some forms of discrimination. If a young female driver manages to drive with care, this will be reflected in her premium, and she may not just be discriminated against on the basis of age.^99^ Barry and Charpentier also make the case that the behavioral data gathered by telematics devices are more reliable than demographic data.^100^ However, telematics and the associated data do bring with them more general fairness issues around surveillance, autonomy, and privacy. More specifically, there is a risk that drivers will feel forced to cede data to motor insurance in order to access fairly priced insurance, which itself raises a question on consumer autonomy.^101^

This discussion on telematics does raise issues that also are of some importance to the developments in the health insurance market. By way of further context, as part of the move to automated vehicles, we are likely to move through a phase of technological development where the driving task is shared and where the vehicle will gather biometric data to assess the readiness of the human driver to resume control.^16^^,^^68^ Thus, we will see a convergence in the type of data utilized in the motor insurance market and the modern health insurance practices that use data from such devices as Fitbit. This will introduce new data regimes where health-based characteristics such as cognitive abilities (for example, the ability to concentrate) are part of insurance calculations. In the main, telematics is focused on behavior, whereas the data gathered on levels 3 and 4 of automation will include health status. This clearly has an ethical dimension and could, for example, result in certain cohorts of older drivers becoming uninsurable. The inability to purchase motor insurance would have negative effects in terms of mobility and employability.^1^ Similar concerns around the wider socio-economic effects exist around device-derived health insurance data where high-risk groups could be excluded or priced out of life insurance.

### What is the role of data ethics in the context of insurance?

The term data ethics is relatively recent and dates from the first years of the twenty-first century. Its roots reside with the term information ethics, which, like its successor, is a hybrid of disciplines including philosophy, computer science, and the social sciences. It concerns itself with the interface of the human with the data realm and the need to preserve human dignity and manage to change power relations.^102^ Implicit here are the related concepts of access, transparency, fairness, equality, and justice that are more commonly framed in terms of bias and non-discrimination. More recently, in terms of industry and the academy, we have seen the advent such of concepts as ethically aligned design, value alignment, and corporate digital responsibility (CDR) to mitigate risks posed by the commercial use of big data. In the case of CDR, this

presupposes that ensuring the ethical design and uses of digital technologies and related data is not solely a technological challenge (e.g., developing algorithms for ethical reasoning). Rather, it requires organisations to develop a comprehensive, coherent set of norms, embedded in their organisational culture, to govern the development and deployment of digital technology and data (See Lobschat, L., Mueller, B., Eggers, F., Brandimarte, L., Diefenbach, S., Kroschke, M., and Wirtz, J., 2021. Corporate digital responsibility. Journal of Business Research. 122, 875–888).

As Lobschat et al. suggest, there is a need for industry to internalize the need for the ethical reflection around the use and indeed misuse of digital technologies.^103^ One of the more imminent challenges relates to how to create sufficiently robust governance structures to achieve this aim and whether or not bespoke entities will be required to address ethical concepts such as fairness and non-discrimination. Such CDR-related values and norms share some principles and goals with corporate social responsibility (CSR). CSR encompasses the economic, legal, and ethical expectations that society has of organizations at a given point in time, and we propose that a similar perspective is inherent to any considerations of CDR as well.

### Transparency and explainability

There are also the immanent problems of transparency and explainability. These are not new problems for the insurance industry or indeed for the area of financial services in general. The dilemma of information asymmetry is well documented and not unrelated to the vexed issue of mis-selling, which has dogged parts of the European insurance industry over the last decade.^104^, ^105^, ^106^ The new data asymmetry has become one that offers significant commercial opportunities. Indeed, given the global competitive market, the transformation is no longer optional. Nonetheless, the data ethical consequences may appear an unwelcome appendage in the context of commercial competitiveness, but we maintain the accurate application of data ethics can revitalize the core social benefits of insurance by supporting a balance of innovation informed by accurate assessments of social relations. This can be achieved by enabling transparency and explainability as necessary features to increase the scalability and accessibility of insurance products.

Conceptually, both explainability and transparency are related to fairness and non-discrimination as they are, in many respects, necessary prerequisites. Fairness here relates to the ethical concept of distributive fairness, which is not just about whether someone is offered insurance but about whether the allocation of premium costs across a community of customers is fair. Non-discrimination means discrimination is based on just relevant risk factors and not on protected characteristics such as gender, race, or ethnicity. To demonstrate fairness and non-discrimination requires that AI algorithms should be transparent and explainable. Transparency means that the information content and nature of the algorithm are visible. The Financial Conduct Authority (FCA) distinguishes between model transparency and process transparency.^107^ Model transparency is defined as the model code and the nature of the input-output relationships. Process transparency is about the development and use of the algorithm. We would add the dataset used to train the algorithm and the use of customer information to the disclosure of AI transparency because the training data and customer information are in many ways of equal or greater importance than the algorithm itself.

Explainable AI refers to the mechanism of how the logic and rationale of the AI system are communicated to the customer.^108^, ^109^, ^110^, ^111^ There is increasing interest in explainability in the context of AI and the digital economy.^112^^,^^113^ For insurance products, which are often underpinned by complex processes, the advent of AI and BDA does present further risks around how consumers understand pricing and the availability of insurance products. Explainability is seen as a key building block in the construction of the ethical use of big data and AI and offers mitigating effects around power and information asymmetries. Note that there is some debate about the distinction between explainability and interpretability.^114^ In this paper, the term explainability refers to the communication from the owner of the AI system to stakeholders, and interpretability is concerned with the social interpretation of that explanation within the specific context of that individual/stakeholder.

How do explanations work is a key starting point in thinking about AI and digital ethics more generally. For Lewis,^115^

To explain an event is to provide some information about its causal history. In an act of explaining, someone who is in possession of some information about the causal history of some event — explanatory information, I shall call it — tries to convey it to someone else.

Causation then is central. For instance, in the context of insurance, explaining to an individual consumer why she or he was denied cover requires satisfying the why question, which involves explaining cause. When we add in a layer of digital technology into sets of causation, explanatory tasks become more difficult. There are black box elements to AI and BDA, which may make causation opaque and difficult to establish.

The configuration and nature of stakeholders around the insurance industry is an important consideration. Kuo and Lupton posit a process for explaining the use of machine learning in insurance pricing, but it is directed at regulators and other insurance professionals.^20^ The ability to generate interpretable AI/machine learning models for regulators is necessary. That said, the degree to which other stakeholders are able to understand the cause of a decision is likely to vary quite widely. Explainability and transparency are functions that are likely to be shared between different entities with insurers, supervisors, and civil society groups all involved.

For insurance consumers, explanations may function best when contrastive; that is to say, when they are sought in the context of other counterfactual cases. To give a more concrete case, why is a particular risk not deemed acceptable instead of being insurable? As Miller points out, explanation is both a process and a product, and why people ask for explanations is an important consideration.^116^ There is a consensus that both transparency and explainability are necessary for trust, which goes to the heart of good governance in financial services. Insurance pricing and risk selection are such that absolute transparency may not always be possible, but some degree of explainability is necessary for maintaining trust and assisting in keeping fairness at the heart of the operation of the insurance industry.

### A hierarchical ethics model of AI systems and big data in insurance

A major barrier in making sense of AI insurance systems is defining what is actually meant by AI and big data in an insurance context. The AI system is often treated as a discrete technical system or even as a black box, which presents an almost intractable problem because it is so complicated and therefore difficult to explain. However, it is possible to define this new wave of technology in conceptual terms that make it much more amenable to analysis and discussion with respect to ethics. In part, this paper is driven by a desire to situate more accurately efforts toward the governance and ethical oversight of AI and big data analysis. We posit the context here as being constituted by four levels: at a product-market level, i.e., the insurance market; at the level of InsurTech, which is embedded within the operations of insurance companies; at the level of human-like AI capabilities, which underpin InsurTech; and lastly at the level of the AI system itself. The last level addresses the need for a detailed examination of how algorithms function and are trained by big data and facilitate the exploration of phenomena such as algorithmic bias. The proposed hierarchical model of AI in insurance is shown in Figure 1.

The structure of the model is based on the logic of a system where the high-level insurance product can be conceptualized as a set of sub-systems that can, in turn, be broken down into more detailed components. This idea of complex systems, composed of organizational and technical systems in a multi-level structure, is central to the engineering discipline.^117^ In this paper, insurance is conceptualized as a complex system that incorporates complex relations across technical and social systems. A systems view is analogous to the levels of abstraction viewpoint of Floridi.^62^ The idea of appealing to layers and levels is common in elucidating different ethical contexts of technological applications and use cases. It has, in recent times, in the context of socio-technological and ethical analysis, become popularized by Floridi^62^ but speaks to a wider methodological discourse.^118^^,^^119^

The broad argument for taking this approach is that, to understand insurance and the ethical issues related to it, it is necessary to be explicit about the definition of insurance products and services, including the specific role of digital and AI technologies and the use of BDA. It is therefore inadequate to simply consider ethical issues using the high-level product-marketing concept of insurance (level 4), and, instead, it is necessary to explore the sub-systems or layers beneath the product to understand the detailed mechanisms of how the technical and social systems interact to create an insurance service.

There are four levels in the model: (1) AI technology, (2) AI capabilities, (3) insurance technology, and (4) insurance products and services. The conceptual model can be applied in a top-down manner, starting with an insurance service, or in a more inductive manner, starting with the AI technology. Taking an inductive approach, the AI technology described in terms of algorithms and data is the core of modern insurance service and represents the design and intent of the overall system. This level includes the digital technology, sources of data, the initial AI design, the process of training the algorithm, and the related digital technologies necessary to locate and instantiate the core AI technology within a broader digital and business context.

Fundamentally, AI technologies create capabilities that are expressed as skills, which are shown in level 2 of the model. AI capabilities are described in terms of human-behavior characteristics, which then form the basis for insurance-specific technology shown in level 3. Insurance technology is a broad term that captures component business processes and services that are recognizable to insurance professionals and customers alike. Insurance technology comprises discrete insurance processes such as smart policy records management, or the development of a risk score, and component insurance services such as robo-advice, accident prevention, and e-service for policy amendments. For example, e-services connect customers with the insurance company and allow them to manage their ongoing relationships by amending their policies, renewing services, and making claims. Pricing is a core insurance activity and can be defined as a distinct business process, which is underpinned by AI capabilities in level 2 and core AI technology in level 1.

Insurance products and services in level 4 are based on an arrangement of insurance technologies to create recognizable insurance products such as car, house, and health insurance. These component processes and basic insurance services can be applied to all forms of insurance and create the building blocks for insurance products, which is the highest level in the model. Insurance products and services define insurance in product-marketing language; that is, insurance products that cover health, automotive, and property, which can be bought and used by consumers in a competitive market.

The importance of the model is 2-fold. First, it relates AI technology and BDA to insurance products through the intervening levels of AI capabilities and insurance technology. Second, it is immediately apparent that ethical issues can be better understood by relating them to specific level(s) in the model. This allows any ethical analysis to be context aware, which speaks to one of the key messages we wish to communicate in this paper; namely, the importance of the relations that exist around the AI (Table 2). A brief overview of different types of ethical issues and how they can be related to each of the levels in the model is described in Table 1.

## Discussion

The model has been illustrated with a range of typical insurance applications and related ethical problems and dilemmas. Each problem has been situated at the most appropriate level to generate insight into the nature of the ethical issues and the likely approaches to resolving or at least mitigating them. Of course, most ethical problems are multi-level problems, but there is usually an obvious starting point that corresponds most closely to the nub of the issue. Similarly, insurance exclusion is best understood by focusing on pricing outcomes to explore how this affects different segments or even individuals from an insurance population.

The multi-level concept is important because it illustrates the complexity of ethical issues in insurance while also providing a mechanism to analyze the situation in a systematic and logical manner. There is also the additional factor of stakeholders because the ethical issues may look quite different from the perspective of a regulator versus a customer.

An example of a multi-level problem from the perspectives of different stakeholders is the difficult issue of transparency and explainability. For a regulatory body that is interested in the detailed mechanisms of a risk model, a comprehensive evaluation of AI technology (level 1) and an explanation of how the outputs from the AI algorithm are subsequently used for analytics (level 2), pricing (level 3), and personalized marketing (level 4) would be vital. For a consumer, it could be adequate to be given an overview of the logic of the algorithm and the type of data that are required (level 1); the logic of the pricing model (level 3), e.g., based on a driving safety score; or a counterfactual explanation that illustrates how changes in customer attributes and behavior change the pricing, and to experience the product through a continued communication (level 4). The general point here is that the multi-level structure provides a framework for analyzing and evaluating ethical problems from a purely technical perspective on level 1 all the way through to the product-market level, where the interactions between the insurance provider and the customer take place. It also allows a focus on a particular insurance technology, such as accident prevention or e-services, and can be employed by different stakeholder groups.

Although we posit the value of understanding specific contexts and the importance of a particular set of relations, there is a need to keep in view the more macro elements in terms of more general impact. Procedural fairness is concerned with whether the overall design, captured by the concept of procedures, and whether the design is applied equally to everyone. In terms of the model, procedural fairness is defined by the way in which the AI technology level operates, how the AI capabilities are applied to solve a particular insurance problem, the manifestation of AI capabilities in core insurance processes, and the use of the service in a market. That is, procedural ethics is best described as a multi-level problem because the description and explanation of a set of procedures depend on an understanding of how each of the levels in the model operates independently and also in terms of how they interact with each other to generate an outcome.

The use case here is insurance, but the hierarchical model can be applied more generally to business ethics problems because the ethical issues and solutions, or at least methods to mitigate them, can be explored using the four levels, which are generic to the design and use of AI systems in a business context. It can also be used by different stakeholders.

### Some conclusions

This piece speaks to wider debates on technological ethics, which includes, among others, Sheila Jasanoff,^120^ Luciano Floridi,^59^^,^^61^^,^^121^ and, of course, Shannon Vallor.^26^ Our article offers a specificity born out of questions regarding BDA and AI in insurance business models and EIOPA's work on digital ethics in insurance. Both combine to offer an empirical basis for discussion. The expert group examined not only the fairness concept around pricing and underwriting but also the necessary governance regimes that will need to be put in place to safeguard the interests of stakeholders. This, to some extent, chimes with both virtue ethics perspectives and that of relational ontology-based approaches as both demand an understanding of context. The contextual element of the paper is also expanded in the proposed hierarchical ethics model, which illustrates how the core AI technology is developed through a series of levels to result in a product-market context where the insurance services are used and where the interactions between insurance firms and customers take place.

If we consider these issues from a *task* perspective (in this case, the regulation of financial services), it is clear that different ethical theories do offer degrees of usefulness. For example, the utilitarian perspective complements risk- or harm-based approaches in the regulation of the insurance industry, while the focus on conduct in the industry does put one in mind of virtue ethics. Somewhat paradoxically, perhaps, this quite specific case of the need for ethical behavior set around the introduction and use of BDA and AI in insurance points to the need for inclusive methods that transcend traditional schools of thought. A focus on the tasks of the EIOPA Expert Group on Digital Ethics brings into relief the distinct elements that are required for better and more ethically informed decision making in high-value data-centered industries.

As a group, we were aware of the need for better explainability in order to flatten power relations between consumers and financial institutions and tackle the information asymmetries. Governance was seen as an important element in terms of process and responsibility. There remains a particular challenge around the issue of agency and conduct. The creation of AI and BDA systems is a distributed task that includes different types of insurance professionals, technology, and data specialists. The systems and the outputs can be somewhat opaque both within insurance organizations and to regulators and other stakeholders. Given the profundity of the changes being brought about, there may be a need for novel forms of oversight, perhaps even the introduction of bespoke digital ethics committees. That said, it is evident that a strategic governance regime combining several governance instruments is the most optimal response. A strategy could involve supporting more informed decisions for stakeholders developing and delivering insurance products and supporting more informed decision making for customers. The industry could achieve this by supporting more transparency and explainability regarding machine decisionality, automation, risk profiling, and customer data in pricing and delivering insurance products and services.

## Experimental procedures

### Resource availability

#### Lead contact

Dr. Martin Cunneen.

#### Materials availability

No materials are available for this work.

#### Data and code availability

There is no data or code availability to declare for this work.
